# Supplementary material for: No obligatory trade-off between the use of space and time for working memory
Source: Commun Psychol. 2023 Dec 14;1:41. doi: 10.1038/s44271-023-00042-9 (PMC11041649; doi:10.1038/s44271-023-00042-9)
Supplement: Supplementary file 1 — Reporting Summary [file 44271_2023_42_MOESM1_ESM.pdf]

nature portfolio

Corresponding author(s): Eelke de Vries
Freek van Ede

Last updated by author(s): Nov 2, 2023

Reporting Summary

Nature Portfolio wishes to improve the reproducibility of the work that we publish. This form provides structure for consistency and transparency in reporting. For further information on Nature Portfolio policies, see our Editorial Policies and the Editorial Policy Checklist.

Statistics

For all statistical analyses, confirm that the following items are present in the figure legend, table legend, main text, or Methods section.

n/a	Confirmed	
☐	☒	The exact sample size (*n*) for each experimental group/condition, given as a discrete number and unit of measurement
☐	☒	A statement on whether measurements were taken from distinct samples or whether the same sample was measured repeatedly
☐	☒	The statistical test(s) used AND whether they are one- or two-sided<br>*Only common tests should be described solely by name; describe more complex techniques in the Methods section.*
☒	☐	A description of all covariates tested
☐	☒	A description of any assumptions or corrections, such as tests of normality and adjustment for multiple comparisons
☐	☒	A full description of the statistical parameters including central tendency (e.g. means) or other basic estimates (e.g. regression coefficient) AND variation (e.g. standard deviation) or associated estimates of uncertainty (e.g. confidence intervals)
☐	☒	For null hypothesis testing, the test statistic (e.g. *F*, *t*, *r*) with confidence intervals, effect sizes, degrees of freedom and *P* value noted<br>*Give P values as exact values whenever suitable.*
☐	☒	For Bayesian analysis, information on the choice of priors and Markov chain Monte Carlo settings
☒	☐	For hierarchical and complex designs, identification of the appropriate level for tests and full reporting of outcomes
☐	☒	Estimates of effect sizes (e.g. Cohen's *d*, Pearson's *r*), indicating how they were calculated

Our web collection on statistics for biologists contains articles on many of the points above.

Software and code

Policy information about availability of computer code

Data collection	Matlab R2020a, Psychtoolbox-3.0.16, EyeLink Software by SR research
Data analysis	Matlab R2020a, FieldTrip 20220628, RStudio 2022.07.2

For manuscripts utilizing custom algorithms or software that are central to the research but not yet described in published literature, software must be made available to editors and reviewers. We strongly encourage code deposition in a community repository (e.g. GitHub). See the Nature Portfolio guidelines for submitting code & software for further information.

Data

Policy information about availability of data

All manuscripts must include a data availability statement. This statement should provide the following information, where applicable:

- Accession codes, unique identifiers, or web links for publicly available datasets
- A description of any restrictions on data availability
- For clinical datasets or third party data, please ensure that the statement adheres to our policy

Raw data and analysis code have been made publicly available via the Open Science Framework and can be accessed at https://osf.io/86atz/.

Human research participants

Policy information about studies involving human research participants and Sex and Gender in Research.

Reporting on sex and gender	Gender, age and handedness were self-reported by the participants.
Population characteristics	Healthy human volunteers aged between 18 and 40 years old with no history or neurological or psychiatric conditions.
Recruitment	Participants were recruited through social media and an online participant database (SONA) at the Vrije Universiteit Amsterdam.
Ethics oversight	Ethics committee of the Faculty of Behavioural and Movement Sciences, Vrije Universiteit Amsterdam

Note that full information on the approval of the study protocol must also be provided in the manuscript.

Field-specific reporting

Please select the one below that is the best fit for your research. If you are not sure, read the appropriate sections before making your selection.

☐ Life sciences ☒ Behavioural & social sciences ☐ Ecological, evolutionary & environmental sciences

For a reference copy of the document with all sections, see nature.com/documents/nr-reporting-summary-flat.pdf

Behavioural & social sciences study design

All studies must disclose on these points even when the disclosure is negative.

Study description	Within-subject design with quantitative data.
Research sample	We conducted two experiments with 25 healthy human volunteers in each.
Sampling strategy	We used the same a-priori determined sample size for both experiments, and report how our key outcome measure replicated between experiments 1 and 2. Participant recruitment for the two experiments was performed independently. In Experiment 1 (mean age = 27.24 years, age range = 18-39 years, 15 women; 1 left-handed, 1 ambidextrous) two participants were excluded and replaced due to poor eye-tracking quality. In Experiment 2 (mean age = 21.32 years, age range = 18-28 years, 19 women; 2 left-handed) one participant was excluded and replaced due to poor eye-tracking quality.
Data collection	Experiment 2 was collected after Experiment 1. The experimenter was not blind to purpose of the either study. Eye-tracking data was collected with an EyeLink 1000 Plus (SR Research, Ltd.) at a sampling rate of 1000 Hz. The task was presented on a computer screen.
Timing	Data for experiment 1 was colected in 2021. Data for experiment 2 was collected in 2022
Data exclusions	In experiment 1, two participants were excluded and replaced due to poor eye-tracking quality. In experiment 2, one participant was excluded and replaced due to poor eye-tracking quality.
Non-participation	All participants that started with the experiment finished the entire session.
Randomization	Experimental conditions were manipulated within-participant.

Reporting for specific materials, systems and methods

We require information from authors about some types of materials, experimental systems and methods used in many studies. Here, indicate whether each material, system or method listed is relevant to your study. If you are not sure if a list item applies to your research, read the appropriate section before selecting a response.

Materials & experimental systems

n/a	Involved in the study	
☒	☐	Antibodies
☒	☐	Eukaryotic cell lines
☒	☐	Palaeontology and archaeology
☒	☐	Animals and other organisms
☒	☐	Clinical data
☒	☐	Dual use research of concern

Methods

n/a	Involved in the study	
☒	☐	ChIP-seq
☒	☐	Flow cytometry
☒	☐	MRI-based neuroimaging
